# Consumer Evaluation of the Role of Functional Food Products in Disease Prevention and the Characteristics of Target Groups

**DOI:** 10.3390/nu12010069

**Published:** 2019-12-26

**Authors:** Brigitta Plasek, Zoltán Lakner, Gyula Kasza, Ágoston Temesi

**Affiliations:** 1 Department of Food Economics, Faculty of Food Science, Szent István University, Villányi str. 29-43, 1118 Budapest, Hungary; 2 Directorate for Food Safety Risk Assessment, National Food Chain Safety Office, Keleti Károly str. 24, 1024 Budapest, Hungary

**Keywords:** consumer research, willingness to purchase, non-communicable diseases, binary logistic regression, random forest

## Abstract

Our research explores the methods consumers would consider using in order to prevent non-communicable diseases, with consuming functional food products being one of these methods. Previous research has pointed out the importance of missing information such as which diseases worry consumers and what they would use to avoid them. We conducted a personal survey with 1027 people in Hungary about 13 diseases and four prevention methods. We analyzed the results with descriptive statistical methods, binary logistic regression, and random forest. According to our results, the highest proportion of worried respondents think it is justified to consume functional foods for the prevention of digestive problems, a weakened immune system, and high cholesterol level. Our results help to characterize the target group for these three diseases. Completed education plays a key role in choosing functional foods to prevent a weakened immune system. Those with tertiary education are the most likely to choose this prevention method. With the other two diseases, age played a crucial role. All age groups over 36 would be more likely to choose functional foods to prevent digestive problems, whereas in case of high cholesterol level, the 36–50 age group would be more likely to apply this method.

## 1. Introduction

Changes in lifestyle, less physical activity, and improper nutrition have resulted in the spread of non-communicable diseases to the extent that today they pose serious problems, and a significant ratio of deaths are connected to them worldwide [1]. Globally, 41 million people die from non-communicable diseases annually, which constitutes 71% of all deaths. Of these, about 15 million people are in the 30–69 age group. Cardiovascular diseases pose the biggest risk (17.9 million deaths) followed by cancer-related diseases (9.0 million), respiratory diseases (3.9 million), and diabetes (1.6 million) [2].

In Hungary, non-communicable diseases present a host of problems for society. Based on the statistical data of World Health Organization (WHO), it was concluded that in Hungary the average number of years spent in good health was 67.4 years in 2015, and the average life expectancy was 75.9 years [3]. This means that the average Hungarian spends 8–9 years suffering from some disease. Statistical data show that in Hungary, cardiovascular diseases, malignant tumors, chronic respiratory diseases, diabetes, and some other non-communicable diseases accounted for 94% of all deaths in 2016 [4].

Medicaments offer a classic solution to treat diseases. Recently, as a result of regulations, medicaments have become more readily available for consumers, so today they have an array of non-prescription products at their disposal that they can apply to cure themselves [5]. Dietary supplements also offer a widely-used solution; however, these pills play a role in the prevention of diseases already. A survey has shown that elderly consumers consider dietary supplements at least as important as prescription medicine. The same research also concluded that the most common reasons for taking dietary supplements were health protection, disease prevention, and the advice of a health expert [6]. Over the last decades, consumption of dietary supplements has grown exponentially; as Starr [7] demonstrated, in the United States, 4000 products were sold in 1994, and this number grew to more than 90,000 by 2014.

Apart from lifestyle changes and the consumption of dietary supplements, the consumption of functional foods can also reduce the risk of developing diseases. “A food can be regarded as ‘functional’ if it is satisfactorily demonstrated to affect beneficially one or more target functions in the body, beyond adequate nutritional effects in a way that is relevant to either an improved state of health and well-being and/or reduction of risk of disease.“ (p. S26, [8]). The different health claims manufacturers often use to communicate the positive effects of functional foods on health to consumers fall under European Food Safety Authority regulations. The specific regulation is included in regulation (EC) no. 1924/2006 [9]; the EFSA database makes all applicable health claims and nutrition claims available. The EFSA distinguishes different types of claims in the category of health claims, such as “Function health claims” (Article 13 claims), “Risk reduction claims” (Article 14(1)(a)), and “Claims referring to children’s development” (Article 14(1)(b)) [10]. For the purposes of our research, one type of health claim, namely, risk reduction claims, are worth highlighting as they communicate the effect on the reduction of the risk of diseases [10].

In their research in Hungary, Szakály et al. came to the straightforward conclusion that “In the current situation, there is no other choice than to bring the public’s attention to food products that possess an extra nutritional advantage” (p. 16, [11]). Owing to their ingredients, functional food products can help to prevent a variety of chronic diseases [12]. Encouragingly, several studies have indicated that due to increasing consumer concerns related to health, consumers would be willing to change their dietary habits and include products that would have a positive effect on their health in one way or another [13,14]. From the results of their focus group interviews, Szakály et al. concluded that if participants “have to choose between medication and functional food products, all of them would vote for the latter” (p. 23 [15]). 

Due to the complexities of developing functional foods, it is important to assess which diseases consumers are concerned about [16]. However, apart from identifying these diseases, it is also important to examine how consumers would prevent them. Without this information we cannot talk about functional foods developed for, and communicated to, a wide range of consumers, and we cannot expect functional foods to have a bigger role in diets. However, if we are aware of the specific diseases that a wide circle of consumers would choose to prevent with the help of functional foods, then the affected food manufacturers could concentrate on these diseases, and a more remarkable public health effect could be achieved.

At the moment it is not yet known which diseases consumers would seek to reduce the risk of through the consumption of health-protecting food products [17,18]. As Siró et al. put it, “It is more interesting, however, to identify those diseases that consumers would make much account of preventing them by nutrition” (p. 462 [17]). We believe that this missing piece of information is what is needed to get a more complete picture of consumer behavior regarding functional foods. Kraus [19] came closest to answering the question with his research where the potential health effects of functional food products were ranked. Baba et al. [20] maintained that health-related consumer concerns may be a crucial factor in food consumption, and thus also in developing new products. Consumer attitudes to functional food products in light of worrisome diseases have already been investigated in general terms. In accordance with previous research [21,22], Chen’s [23] research results showed that those who were concerned about modern lifestyle diseases displayed a more positive attitude towards functional food products and had a larger likelihood of accepting and using them in order to prevent these diseases. 

The spread of non-communicable diseases poses numerous societal and economic risks. Nevertheless, it is still an unanswered question as to which prevention methods consumers would use to avoid/treat specific diseases, so the aim of the research was to answer this question by asking them.

## 2. Materials and Methods

In our research, we conducted a survey with personal interviews (PPI) with 1027 participants between 4–19 March 2013, at busy transport hubs of five big cities in Hungary, namely in Budapest, Pécs, Sopron, Nyíregyháza, and Szeged. The respondents were rewarded with a small non-food gift for participating in the survey. At the beginning of the survey, participants were informed about the aim of the research; at the same time, the responses were anonymous. Before completing the survey, respondents gave implicit consent to their answers being recorded and later analyzed; the study was conducted in accordance with the Declaration of Helsinki. Respondents had the option to refuse to answer any question or stop answering the survey at any point.

We employed quota sampling, as opposed to probability sampling, so our sample significantly follows the segmentation of society. Among our respondents, compared to the population, the ratio of people between ages 36–50 and >50 is smaller, and the ratio of those living in Budapest and having tertiary education is higher.

In the survey, we asked participants questions in relation to 13 diseases: which are the ones they are not concerned about; the ones they are concerned about but would not make a financial sacrifice to prevent them; the ones they are concerned about and would make a financial sacrifice to prevent them. Moreover, supposing these diseases can be avoided/prevented through various methods, we wanted to find out exactly how much respondents would spend. Demographic variables included gender, age, place of living, highest education, estimated income status of the household, and the presence of underage children in the household. Finally, our questions included whether it was important for the respondents to do something for their health, whether they paid attention to their diet, and who did the grocery shopping in the household.

After collapsing the results, we used the statistics software SPSS 25.0 to analyze the answers. The collected data were first analyzed through descriptive statistical methods, followed by binary logistic regression to find the differences based on demographic variables.

In the course of the regression analysis, we considered only respondents who were concerned and would make a financial sacrifice to avoid the disease under investigation. In the models employed, the dependent variable was the financial expense of the prevention method (purchasing functional food (yes/no or Y/N)) chosen in order to avoid the specified disease, and the independent variables were the characteristics of the respondents, presented in Table 1. In our analyses, we applied the 5% significance level (*p* < 0.05) and the forward Wald method.

The principle of triangulation provided another perspective from which our study approached the research questions related to the identification of diseases that consumers would prevent with the help of functional food products, and the socio-demographic features that have a stronger influence on this choice.

As Meerza et al. (p. 29, [25]) summarized it: “binomial logistic regression generally works well as a classifier when the relationship between dependent and independent variables is linear and the data are relatively balanced between classes [26], the random forest (RF), a machine learning method, outperforms the logistic regression when the non-linear characteristics and complex interactions among predictor variables exist [27]”.

With the aim of forecasting consumer choice, we ran additional statistical analysis on the data. Random forest is a method widely used for making predictions, with the advantages of being wieldy, robust, and fast [28]. We used the random forest package of the R statistical software to examine the extent to which consumer choice can be predicted. The analysis was conducted by including all the examined variables. “Random forests are a combination of tree predictors such that each tree depends on the values of a random vector sampled independently and with the same distribution for all trees in the forest” (p. 5, [29]). During the analysis, we used the “OOB Estimate of Error Rate” value to examine accuracy, and the “MeanDecreaseGini” values to examine the importance of the analyzed variables. With each analysis, 70% of the total sample was used for training and 30% was used to validate the models.

## 3. Results

### 3.1. Identifying the Diseases That Worry Respondents

One of the questions we searched an answer for in our research was to identify the non-communicable diseases that concern consumers the most. Table 2 shows the ratios of the respondents concerned about a specific disease and their propensity to make a monetary sacrifice to avoid it.

According to our results, surveyed consumers are most concerned about cancer-related diseases (78%). This same disease is the one with the highest ratio of respondents willing to make a financial sacrifice. A high ratio of respondents would also make a monetary sacrifice to prevent cardiovascular diseases, a weakened immune system, and diabetes. Surveyed consumers are least concerned about migraines and unbalanced mood.

### 3.2. The Form of Financial Sacrifice Made Towards Preventing Diseases

Our research also aimed at finding out what exactly, if anything, worried respondents would spend on. The four factors we examined were taking medicine, using dietary and nutritional supplements, consuming functional foods, and lifestyle changes. Table 3 summarizes the results of the question of what would worried respondents, who are willing to make a financial sacrifice, spend to avoid a given disease. Respondents were free to indicate more than one answer. In the table we included only the answers of respondents who were worried and willing to make a financial sacrifice to avoid the disease. Thus, the displayed percentages for the prevention methods do not emerge from the whole sample, but from a smaller part of it, which we consider to be 100% here.

Our results show that in order to prevent diseases, most people say that they would implement lifestyle changes. Respondents ascribe the biggest importance to it in the case of cardiovascular diseases, which means that consumers would apply this method to prevent the disease which carries the biggest noncommunicable disease (NCD) risk in Hungary, based on data by WHO [4]. Thus, we can also conclude that consumers are aware of the connection between lifestyle and the emergence of cardiovascular diseases. 

The main aim of our research, however, was to identify the diseases that most people are likely to prevent with the consumption of functional food products. To the question raised by Siró et al. [17], namely, what are the diseases that consumers would prevent by choosing functional foods, our results yielded the following answer: digestive problems, a weakened immune system, and high cholesterol level. These are the three diseases for which consumers would choose functional foods in the highest proportion. If we combine this result with the disease that worries respondents the most, we can conclude that functional food products can play the biggest role in the prevention of a weakened immune system for a wide circle of consumers. It should also be added that functional foods can play an important role in preventing many other diseases apart from the three highlighted in our study, in the case of which a significant reluctance of consumers has to be overcome.

In his research, Kraus [19] ranked the potential health effects of functional food products according to their importance. Consistent with our results, strengthening the immune system and lowering the risk of cancer-related diseases also ranked on top of his list, and improving memory ranked lower.

### 3.3. Disease Prevention with Functional Foods—Consumers’ Characteristics

We considered it important to utilize the characteristics of respondents to identify the groups that can be most associated with specific prevention methods. Table 4 displays the results derived from the binary logistic regression. The table shows only the diseases and characteristics of respondents for which significant differences emerged between the groups within a certain variable. The analyses included all the variables from Table 1. The odds ratios received from the regression analysis show the values compared to the first group of the variable, that is, we can see the results compared to females in the case of gender; compared to the 18–25 age group in the case of age; to the primary/vocational school educated in the case of education; and a different town or village compared to Budapest in the case of place of living. Regarding the question about the person who does the shopping, the basis of comparison is the case when the respondent does the shopping; to this we compare the case when someone else does it or when it is a shared responsibility. In the case of the statement “I pay attention to healthy eating”, we compare those for whom it is important to those for whom it is not. 

The table in Appendix A includes the goodness of fit test of models and the indicators that characterize the explanatory power of the models. According to the results of the Hosmer–Lemeshow test, the goodness of fit of the models is appropriate, and apart from one instance, none of the values is significant. However, based on the two other indicators (Cox–Snell R^2^ and Nagelkerke R^2^), the considered independent demographic variables only partially explain the dependent variables. The Cox–Snell R^2^ values were around 0.05 on average, and the Nagelkerke R^2^ values were around 0.07. From the small values we can conclude that apart from the demographic variables, other factors also influence whether a consumer chooses the prevention method under consideration in order to avoid diseases.

Based on the results displayed in Table 4, males consider the consumption of functional food as relevant prevention methods to a lesser extent in the prevention of cancer, migraine, osteoporosis, and a weakened immune system. Thus, we can emphasize that in the case of these diseases, it is important that companies address their prevention-related communication primarily at women. This result is in line with previous literature [30,31,32,33,34], which state that women are more open to functional food; at the same time, our results provide a more nuanced picture in that they identify the diseases for the prevention of which functional food development should be targeted to women. 

For six of the 13 examined diseases, we obtained a significant result related to the effect of age. The results show that compared to the 18–25 age group, it was the 36–50 age group that yielded significant differences in the highest number. This age group considered functional food products more important in the prevention of high cholesterol level, diabetes, osteoporosis, a weakened immune system, and digestion problems. For this last problem, buying and consuming functional foods can be an important prevention method also for the 50+ age group, whereas for memory and concentration problems, the 25–35 age group may consider this method. This result of ours clarifies the statements of previous literature [17,31,32,34,35], according to which it is the middle-aged and the elderly rather than the young who would consume such products.

It is also important to highlight the aspect of the person who does the grocery shopping, as for three diseases, we obtained a significant result in this respect. For all of the three diseases, namely, cancer-related diseases, skin diseases, and joint problems, it is a shared finding that respondents in the household of whom someone else does the shopping consider the consumption of functional foods a less relevant prevention method than respondents who themselves do the shopping.

Table 4 also includes significant results related to the highest level of completed education of respondents. The responses of those with a higher level of education were compared to those that were primary/vocational school educated. In accordance with results of previous research literature [17,30,31,32,33], those with a college/university education consider the consumption of functional foods more suitable for the prevention of certain diseases. Those with a college/university education consider the consumption of functional foods a suitable solution for the prevention of cancer-related diseases, memory and concentration problems, and a weakened immune system, whereas for the prevention of joint problems, it is rather those with a secondary technical school/secondary school diploma who would choose this method.

For certain diseases, place of living or the emphasis on a healthy diet proved to be an important factor. Place of living gained importance with diabetes. Respondents living in the capital consider the potential functional foods less suitable for its prevention. The importance of a healthy diet may play a main role in the choice of products aimed at preventing a weakened immune system or osteoporosis.

Of all the diseases, we obtained the highest exp (B) value related to a weakened immune system. Our results show that for the prevention of this disease, it is respondents with a higher level of education who consider the consumption of functional foods the most suitable method.

We can make sense of the results in Table 2, Table 3 and Table 4 collectively. Comparing Table 2 and Table 3, we can highlight a weakened immune system, diabetes, and digestive problems as diseases that worry a significant number of respondents (Table 2), for the prevention of which a higher ratio of the worried would also choose functional foods (Table 3). Adding in the results of the binary logistic regression (Table 4) with the characteristics of respondents, the consumer segment which is more likely to consume functional foods in order to prevent specific diseases can be properly outlined.

Thus, for the prevention of a weakened immune system, functional foods are considered the most suitable by women, by those with a higher level of education, by the 36–50 age group, and by those for whom a healthy diet is important. If a product manufacturer would like to communicate the prevention of a weakened immune system—an aim towards which, according to our results, 39% of those concerned and willing to spend would purchase functional food—to its future consumers, then it is important for them to assess how to reach consumers with these characteristics. For the prevention of diabetes, it is the 36–50 age group and those living in a place other than the capital who would give an important role to functional foods. In connection with digestive problems, the over 36 age group can be highlighted, as they consider the consumption of functional foods a relevant prevention method for this disease. 

### 3.4. Random Forest with the Aim of Identifying Factors that Influence Consumer Decision

The results were also analyzed with the random forest method. Our aim was to identify the demographic variables that most influence the decisions of consumers who would choose functional food products to prevent diseases. The analysis was conducted by including all 10 variables. In Table 5, only three variables are presented, namely those with the highest MeanDecreaseGini value. The importance of the other variables is summarized in Appendix B.

The method worked with the lowest error rate in the case of mood disorders/apnoea, migraine, joint diseases, and respiratory diseases for the grouping of respondents and the prediction of their choice of functional food products to prevent diseases. During the statistical analysis, we examined the importance of the 10 variables summarized in Table 1 with the help of MeanDecreaseGini. The age and education of the consumer have a stronger influence during prediction. Age was more important than the other factors for 12 of the 13 examined diseases, whereas in one case, education was more important. Consumers’ place of living emerged among the first three most important influencing factors for nine diseases, whereas in two cases, consumers’ BMI rating and perception of income, and in one case, the identity of the primary grocery shopper, were among the most important influencing factors. Contrary to previous results [30,31,32,33], which found that an important factor in the consumption of functional foods is the gender of the consumer, in our analysis this factor was not among the top three factors influencing prediction for any of the diseases.

During the triangulation process, we also compared the results obtained via the random forest method with those of the regression analysis in order to arrive at a more accurate and justified result in terms of the most relevant target group for disease prevention through functional foods. The ExpB values of the regression analysis and the MeanDecreaseGini values of the random forest analysis complete each other such that the value of importance indicates the most influential factor in the prediction of consumer choice, while the odds ratio makes it more precise.

This information helps to identify the factors worth emphasizing for individual diseases. In the case of weakened immune system, education is the most important factor for making predictions, and based on the regression analysis, those with tertiary education can be an important target group.

In terms of the age of the consumer, five diseases can be highlighted among those for which age was the most important factor when making predictions. During product development and the preparation of marketing communication, companies should focus on the 36–50 age group for the prevention of high cholesterol level, digestive problems, diabetes, and osteoporosis, the 25–35 age group for the prevention of memory lapses and concentration problems and the 36 or older age groups for digestive problems.

## 4. Discussion

In our study we investigated which of the most dangerous non-communicable diseases worry consumers the most and also what exactly people would spend on if they are concerned about a disease and would make a monetary sacrifice to avoid it. We examined 13 diseases and four prevention methods.

The conclusion of our research is that most consumers are aware of the risks of non-communicable diseases and they are worried about them, and, depending on the disease, only a small group of the concerned is not willing to make a financial sacrifice to avoid them.

Our results show that with most diseases, lifestyle change is the most favored prevention method of consumers, followed by, depending on the disease, the consumption of functional foods or dietary/nutritional supplements. Except for a few cases, medication as a treatment method is the least sought-after, and the most divisive option for consumers.

Lifestyle changes are the most preferred methods for most of the diseases, such as for the prevention of cardiovascular diseases and unbalanced mood/apnoea and a high level of cholesterol, while dietary supplements are chosen to prevent a weakened immune system. It is important to point out that with several diseases, the smallest ratio of respondents chose dietary supplements. Further results indicate that consumers are the most concerned about cancer-related diseases. This is the only disease where, besides lifestyle changes, consumers consider taking medicaments as the best option.

Consumers did not consider the consumption of health-protecting food products the best solution in any of the cases. Even if not the best option when trying to prevent/treat diseases, it is still a relevant choice when trying to avoid digestion problems, a weakened immune system, high level of cholesterol, diabetes, and cancer. In his research, Kraus [19] drew up an order of importance regarding the health effects of a product. Some of his results coincide with the order of importance we arrived at. Strengthening the immune system and the prevention of cancer-related diseases as motivating factors for the consumption of functional foods also appeared at the top position in that research, whereas improving memory was ranked lower.

In order to utilize the characteristics of respondents to identify the groups that can be most associated with the specific prevention methods, we conducted binary logistic regression. In the course of the regression analysis, we investigated the 10 examined variables to identify the groups we can emphasize within each variable for each disease. Altogether we obtained multiple significant results in relation to age (the 36–50 age group), completed education (college/university), place of living, and the primary grocery shopper of the household.

Based on the analysis done with the random forest statistical method, we can state that the choice made by consumers participating in the survey can be predicted with an error rate of 26%–47%. Among the examined variables, age and education showed greater importance; also, with some diseases, place of living, BMI rating, and the identity of the primary grocery shopper in the household played an important role in the prediction of decisions.

In accordance with the principle of triangulation, we examined our aim from several perspectives. Comparing the results of the regression and random forest, we can more firmly state that the completed education of respondents plays a bigger role in the prediction of the choice for functional food products in the prevention of a weakened immune system; more specifically, this prevention method is relevant for those with tertiary education. Age plays a more important role in predicting whether respondents purchase functional foods or not to prevent high cholesterol level, digestive problems, diabetes, osteoporosis, memory lapses and concentration problems. For the first four diseases, the 36–50 age group is the relevant target, for memory lapses and concentration problems the 25–35 age group is the relevant target and for digestive problems, the 36 or older age group is the relevant target.

Numerous literature sources [17,30,31,32,33,34,35] emphasize the important role of gender, age, and completed education as key demographic factors influencing the consumption of functional foods. However, by looking at such products from a different angle, apart from age and completed education, the results of our research also confirm the effects of other influencing factors, such as place of living, BMI rating, and the perception of income. Moreover, our results also show that in relation to disease prevention, the gender of the consumer does not have a significant effect on choosing functional foods.

These new pieces of information are filling a long-standing information gap. Our results help functional food manufacturers with the identification of diseases for which the effects of their products are worth emphasizing, and with the identification of consumer target groups. The results of the research are useful for experts working in the field of product development and for marketing communication professionals as well. We recommend that both functional food developmental research and manufacturing companies’ research and development (R&D) departments, as well as their marketing experts devote their resources to developing and communicating about products helping to prevent diseases that have the highest consumer acceptance. It would also help to facilitate all of this if this viewpoint—that the planned functional food development should aim at preventing a disease that consumers would like to prevent by consuming functional foods—was included as a basis for the assessment of international or national grants. This is how we can expect functional food consumption to increase significantly and play a more important role in improving society’s health.

### Limitations

This research is the very first step towards answering a complex question. Due to the limited scope of the analysis, some of the steps in this research were aimed to be simple. Future research should include the analysis of the threat of multi-collinearity as well.

Although the current health conditions of the participants and their family can influence their answer to the given questions, we did not address this problem. Future research could focus on this aspect as well.

The answer we have received is not a general one, as Hungary is a small country in Europe. For a firmer, more widely accepted answer, international research might be useful.

## Figures and Tables

**Table 1:** Grouping of respondents based on variables (*n* = 1027).

Characteristics of Survey Respondents		%
Gender	Female	53.5%
	Male	46.5%
Age brackets	<25	47.7%
	25–35	18.8%
	36–50	14.2%
	50<	19.3%
Place of living	Budapest	39%
	Another city	48.6%
	Village	12.4%
Highest accomplished qualification	Primary school/vocational school	15.1%
	Secondary technical school/secondary school diploma	40.0%
	Tertiary education	44.9%
Subjective estimation of income position	Below average	35.5%
	Average	53.6%
	Above average	10.9%
Presence of a child under 18 in the household	Yes	79.5%
	No	20.5%
Overweight or underweight status of the respondent (based on BMI) *	Undernourished	6.8%
	Normal	61.3%
	Obese/overweight	31.9%
It is important for me to do something for my health	Important	88.9%
	Less important/not important	11.1%
I pay attention to healthy eating	Important	72.8%
	Less important/not important	27.2%
Grocery shopper	Respondent	25.7%
	Another person	22.7%
	Shared	51.6%

* Based on World Health Organization (WHO) body mass index (BMI) [24].

**Table 2:** The attitude of respondents towards different health problems and their propensity to spend money to decrease the probability of their occurrence.

Disease	Concerns, and the Respondent Is Ready to Spend Money to Prevent It	Concerns, but the Respondent Does Not Want to Spend Money to Prevent It	Does Not Concern Respondents
Cancer	57%	21%	22%
Cardiovascular diseases	49%	28%	23%
Weakened immune system	48%	24%	28%
Diabetes	42%	24%	34%
Osteoporosis	38%	26%	36%
Joint diseases	37%	31%	32%
Skin diseases	33%	28%	39%
Digestive problems	33%	33%	34%
High level of cholesterol	32%	33%	35%
Respiratory diseases	32%	32%	36%
Memory lapse/concentration problems	32%	30%	38%
Migraine	26%	27%	47%
Unbalanced mood/apnoea	21%	35%	44%

**Table 3:** The form of financial sacrifice worried respondents are willing to make.

	Concerned, Ready to Make a Financial Sacrifice to Prevent It		Form of Financial Sacrifice			
			Medicine	Dietary Supplement	Functional Food	Lifestyle Changes
	*n*	%	%	%	%	%
Digestive problems	327	100%	13%	29%	43%	55%
Weakened immune system	473	100%	22%	36%	39%	56%
High cholesterol level	322	100%	12%	19%	37%	64%
Diabetes	418	100%	36%	17%	35%	59%
Cancer	552	100%	47%	19%	31%	48%
Osteoporosis	376	100%	38%	24%	30%	40%
Cardiovascular disease	480	100%	25%	15%	29%	70%
Joint diseases	368	100%	30%	26%	27%	48%
Skin diseases/eczema	325	100%	47%	14%	26%	37%
Memory lapses/concentration	317	100%	25%	26%	26%	46%
Mood disorders/apnoea	211	100%	12%	21%	24%	66%
Respiratory diseases	311	100%	39%	15%	21%	47%
Migraine	253	100%	43%	11%	21%	44%

**Table 4:** Results of the binary logistic regression.

Number of Cases	Characteristics of the Respondent (Independent Variable)	Level of Attribute	Would Buy Functional Food to Avoid the Specified Disease (Dependent Variable)						
			B	SE	Wald	Sig.	Exp(B)	95% CI for EXP (B)	
								Lower	Upper
High cholesterol level									
289	Constant		−0.752	0.189	15.773	0.000	0.471		
	Age	36–50	0.919	0.346	7.056	0.008	2.508	1.273	4.942
Cancer-related diseases									
498	Constant		−0.745	0.371	4.024	0.045	0.475		
	Gender	Men	−0.469	0.208	5.108	0.024	0.626	0.416	0.940
	Level of education	College/university	0.992	0.356	7.749	0.005	2.697	1.341	5.423
	Grocery shopper	Another person	−0.862	0.296	8.498	0.004	0.422	0.236	0.754
Skin diseases									
293	Constant		−0.455	0.251	3.301	0.069	0.634		
	Grocery shopper	Another person	−0.743	0.362	4.205	0.040	0.476	0.234	0.968
		Shared	−0.797	0.321	6.170	0.013	0.451	0.240	0.845
Memory lapse/concentration problems									
284	Constant		−1.871	0.483	15.006	0.000	0.154		
	Age	25–35	0.875	0.359	5.933	0.015	2.399	1.186	4.852
	Level of education	College/university	1.004	0.494	4.132	0.042	2.728	1.037	7.181
Digestion problems									
291	Constant		−1.103	0.396	7.758	0.005	0.332		
	Age	36–50	1.055	0.362	8.478	0.004	2.872	1.412	5.844
		50+	0.940	0.332	8.013	0.005	2.559	1.335	4.906
Migraine									
224	Constant		−0.927	0.197	22.183	0.000	0.396		
	Gender	Men	−1.129	0.376	9.022	0.003	0.323	0.155	0.675
Diabetes									
375	Constant		−1.247	0.240	26.923	0.000	0.287		
	Age	36–50	0.997	0.330	9.121	0.003	2.710	1.419	5.177
	Place of living	Another city	0.509	0.252	4.074	0.044	1.664	1.015	2.729
		Village	0.984	0.357	7.588	0.006	2.674	1.328	5.385
Joint diseases									
329	Constant		0.118	0.402	0.086	0.770	1.125		
	Level of education	Secondary technical school/secondary school diploma	−0.981	0.408	5.783	0.016	0.375	0.168	0.834
	Grocery shopper	Another person	−0.956	0.379	6.351	0.012	0.384	0.183	0.809
Osteoporosis									
333	Constant		−1.495	0.345	18.827	0.000	0.224		
	Gender	Men	−0.518	0.254	4.161	0.041	0.596	0.362	0.980
	Age	36–50	1.087	0.347	9.816	0.002	2.966	1.502	5.856
	I pay attention to healthy eating	Important	0.875	0.345	18.827	0.012	2.398	1.209	4.758
Weakened immune system									
431	Constant		−1.881	0.439	18.325	0.000	0.152		
	Gender	Men	−0.472	0.213	4.925	0.026	0.624	0.411	0.946
	Age	36–50	0.938	0.314	8.916	0.003	2.554	1.380	4.727
	Level of education	College/university	1.355	0.381	12.656	0.000	3.875	1.837	8.174
	I pay attention to healthy eating	Important	0.616	0.266	5.358	0.021	1.852	1.099	3.121

**Table 5:** Results obtained with the help of random forest.

Disease	OOB Estimate of Error Rate	Relative Importance	
			MeanDecreaseGini
Cardiovascular disease	33.66%	age	23.52
		grocery shopper	19.92
		education	19.40
High cholesterol level	40.89%	age	27.12
		education	22.02
		subjective estimation of income position	21.54
Cancer	38.11%	age	21.88
		education	20.85
		place of living	19.60
Mood disorders/Apnoea	26.15%	age	20.15
		place of living	17.57
		education	17.19
Respiratory diseases	31.15%	age	20.74
		education	18.90
		place of living	18.22
Skin diseases, eczema	33.38%	age	24.67
		education	19.89
		place of living	19.89
Memory lapses, concentration	34.08%	age	22.18
		education	19.78
		place of living	18.56
Digestive problems	46.73%	age	28.10
		place of living	24.55
		education	23.90
Migraine	28.09%	age	20.34
		education	16.70
		BMI category	16.57
Diabetes	45.48%	age	24.66
		place of living	22.16
		subjective estimation of income position	21.48
Joint diseases	30.88%	age	23.96
		education	21.71
		BMI category	20.18
Osteoporosis	36.68%	age	24.53
		education	19.66
		place of living	19.32
Weakened immune system	39.64%	education	23.12
		age	23.00
		place of living	21.40

Notes: 70% of the sample was used to train, 30% was used to validate the models; accuracy, sensitivity and specificity values of models can be found in Appendix C.

## Appendix A

**Table A1:** Goodness of fit tests of model.

Would Buy Functional Food to Avoid the Specific Disease	Hosmer–Lemeshow Test (*p*-Value)	Cox–Snell R^2^	Nagelkerke R^2^
High cholesterol level	1.000	0.027	0.036
Cancer-related diseases	0.880	0.059	0.082
Skin diseases	1.000	0.023	0.033
Memory lapse/concentration problems	0.892	0.064	0.093
Digestion problems	0.951	0.074	0.099
Migraine	-	0.044	0.069
Diabetes	0.014	0.052	0.071
Joint disease	0.991	0.040	0.057
Osteoporosis	0.997	0.073	0.102
Weakened immune system	0.913	0.102	0.138

## Appendix B

**Table A2:** Further results obtained with the help of random forest.

Disease	OOB Estimate of Error Rate	Relative Importance	
			MeanDecreaseGini
Cardiovascular disease		Place of living	18.98
		Gender	13.41
		Presence of a child under 18 in the household	9.98
		Subjective estimation of income position	18.95
		It is important for me to do something for my health	7.73
		I pay attention to healthy eating	10.20
		BMI category	19.34
High cholesterol level		Place of living	20.95
		Gender	14.04
		Presence of a child under 18 in the household	11.63
		Grocery shopper	18.62
		It is important for me to do something for my health	8.50
		I pay attention to healthy eating	12.51
		BMI category	20.21
Cancer		Gender	11.85
		Presence of a child under 18 in the household	11.74
		Grocery shopper	18.59
		Subjective estimation of income position	19.83
		It is important for me to do something for my health	7.71
		I pay attention to healthy eating	11.61
		BMI category	18.90
Mood disorders/apnoea		Gender	9.29
		Presence of a child under 18 in the household	9.91
		Grocery shopper	15.55
		Subjective estimation of income position	14.61
		It is important for me to do something for my health	7.06
		I pay attention to healthy eating	9.45
		BMI category	16.66
Respiratory diseases		Gender	12.54
		Presence of a child under 18 in the household	11.13
		Grocery shopper	17.81
		Subjective estimation of income position	17.54
		It is important for me to do something for my health	8.09
		I pay attention to healthy eating	11.02
		BMI category	16.42
Skin diseases/eczema		Gender	13.10
		Presence of a child under 18 in the household	11.26
		Grocery shopper	17.96
		Subjective estimation of income position	16.37
		It is important for me to do something for my health	7.68
		I pay attention to healthy eating	11.05
		BMI category	19.29
Memory lapses/concentration		Gender	12.15
		Presence of a child under 18 in the household	11.65
		Grocery shopper	17.54
		Subjective estimation of income position	17.64
		It is important for me to do something for my health	8.31
		I pay attention to healthy eating	10.98
		BMI category	17.87
Digestive problems		Gender	15.24
		Presence of a child under 18 in the household	11.90
		Grocery shopper	22.28
		Subjective estimation of income position	22.65
		It is important for me to do something for my health	9.65
		I pay attention to healthy eating	11.22
		BMI category	21.93
Migraine		Place of living	16.51
		Gender	12.04
		Presence of a child under 18 in the household	9.34
		Grocery shopper	15.10
		Subjective estimation of income position	15.43
		It is important for me to do something for my health	7.33
		I pay attention to healthy eating	10.01
Diabetes		Gender	13.82
		Presence of a child under 18 in the household	12.48
		Grocery shopper	20.31
		Subjective estimation of income position	21.48
		It is important for me to do something for my health	9.21
		I pay attention to healthy eating	12.44
		BMI category	19.70
Joint diseases		Place of living	18.70
		Gender	13.39
		Presence of a child under 18 in the household	11.81
		Grocery shopper	17.88
		Subjective estimation of income position	17.87
		It is important for me to do something for my health	8.88
		I pay attention to healthy eating	11.68
Osteoporosis		Gender	12.90
		Presence of a child under 18 in the household	11.75
		Grocery shopper	18.58
		Subjective estimation of income position	17.93
		It is important for me to do something for my health	8.80
		I pay attention to healthy eating	10.15
		BMI category	17.36
Weakened immune system		Gender	13.69
		Presence of a child under 18 in the household	12.29
		Grocery shopper	20.62
		Subjective estimation of income position	20.45
		It is important for me to do something for my health	9.98
		I pay attention to healthy eating	12.27
		BMI category	19.13

## Appendix C

**Table A3:** Accuracy, sensitivity, and specificity of random forest models.

Disease	Accuracy	Sensitivity	Specificity
Cardiovascular disease	0.6861	0.9227	0.1011
High cholesterol level	0.6246	0.8557	0.1944
Cancer	0.6214	0.88835	0.08738
Mood disorders/apnoea	0.7184	0.97778	0.02381
Respiratory diseases	0.7152	0.93191	0.02703
Skin diseases/eczema	0.7023	0.94619	0.06977
Memory lapses/concentration	0.6634	0.8711	0.1071
Digestive problems	0.5372	0.7596	0.2143
Migraine	0.7379	0.9619	0.0137
Diabetes	0.6375	0.8702	0.1584
Joint diseases	0.6828	0.9178	0.1111
Osteoporosis	0.6204	0.8820	0.1354
Weakened immune system	0.5761	0.8939	0.1385
